# The more academic burnout students got, the more problematic mobile phone use they suffered? A meta-analysis of mainland Chinese adolescents and young adults

**DOI:** 10.3389/fpsyg.2022.1084424

**Published:** 2023-01-16

**Authors:** Shunyu Li, Mengmeng Xu, Yuxuan Zhang, Xiaotong Wang

**Affiliations:** ^1^Center for Higher Education Developmet Research in Xinjiang, Xinjiang Normal University, Urumqi, Xinjiang Uygur Autonomous Region, China; ^2^Department of Social and Behavioural Sciences, City University of Hong Kong, Kowloon, Hong Kong SAR, China; ^3^School of Education, Liaoning Normal University, Dalian, Liaoning Province, China

**Keywords:** academic burnout, problematic mobile phone use, mainland China, adolescents and young adults, meta-analysis

## Abstract

**Introduction:**

In recent years, the relationship between academic burnout (AB) and problematic mobile phone use (PMPU) has become the hot issue of scholars, and a lot of related research has been carried out, but the results are different. Most studies showed that there was a significant positive correlation between AB and PMPU. However, some studies showed that the relationship between AB and PMPU was not significant. While this study aimed at exploring the relationship between AB and PMPU, as well as the influence of some moderating factors (region, age, gender, publication year, the measurement instrument) on them.

**Methods:**

According to the searching process for studies of PRISMA, we searched the related studies on AB and PMPU in Mainland China from January 2012 to November 2022 from CNKI, Wanfang Data, Chongqing VIP Information Co., Ltd. (VIP), Baidu scholar, ProQuest dissertations, Taylor & Francis, Springer, Web of Science, Google Scholar, EBSCO and PsyclNFO. Eventually, 50 studies were included in the meta-analysis, involving 38,488 subjects, with the sample size ranging from 193 to 2,260. CMA 3.0 was used to analyze the overall effect and test the moderating effect.

**Results:**

The result shows that the relationship between AB and PMPU can be considered as a moderate correlation (*r* = 0.414, 95%CI [0.384, 0.443]), and moderator analysis shows that the relationship between AB and PMPU was moderated by the measurement instrument and publication year.

**Discussion:**

Specifically, when the Mobile Phone Addiction Tendency Scale and the Smartphone Addiction Scale for College Students were used as PMPU measurement tools, the correlation coefficients between AB and PMPU were higher. When the publication year was used as a moderating variable, the relationship between AB and PMPU increased over the years.

**Systematic Review Registration**: https://www.crd.york.ac.uk/prospero/display_record.php?ID=CRD42022347277, identifier PROSPERO CRD42022347277.

## Introduction

As of June 2022, the number of mobile Internet users in China was 1.047 billion, accounting for 99.6% of the total Internet users. The popularity of mobile phones has brought a lot of convenience to people’s life. People can use mobile phones to communicate, as well as getting news and making online payment, webcast and car booking, etc. [CNNIC, 2022]. However, excessive use of mobile phones brings about some new social problems, such as Problematic Mobile Phone Use (PMPU).

PMPU is also termed mobile phone addiction, mobile phone overuse, and mobile phone dependence (Wang C. et al., 2021; Zhong et al., 2021; Liu Q. et al., 2022). Scholars did not get a consensus on the definition of PMPU, which can be generally divided into two types. On the one hand, PMPU was treated as addictive behavior. For example, based on the addiction literature, especially technological and behavioral addiction literature, Bianchi and Philips developed the Mobile Phone Problem Usage Scale (MPPUS) with internal consistency reliability of 0.93 (Bianchi and Phillips, 2005). The MPPUS includes tolerance, withdrawal, escaping from other problems, craving, and negative life situations in the areas of social, working, familial, and financial difficulties (Bianchi and Phillips, 2005). The publication of this questionnaire has laid a foundation for subsequent research. Leung, a scholar from Hong Kong, China, believed that mobile phone addiction was an impulse control disorder, similar to the characteristics of pathological gambling (Leung, 2008). Based on MPPUS and referring to Young’s measurement criteria for Internet addiction and the criteria for assessing substance dependence in the Diagnostic and Statistical Manual of Mental Disorders (DSM-IV), Leung developed the mobile phone addiction index (MPAI) for adolescents (14–20 years old). The scale with 17 items consists of four dimensions: inability to control craving, withdrawal and escape, feeling anxious and lost, and productivity loss. The internal consistency coefficient of this scale is 0.90 (Leung, 2008). Besides, Xiong compiled the Mobile Phone Addiction Tendency Scale (MPATS) by referring to MPPUS and the primary symptoms of Internet addiction proposed by Young. The scale focused on the mobile phone users’ internal processing activities and subjective experience of social interactions, which is suitable for college students in mainland China. The MPATS’ coefficient of internal consistency is 0.83. The scale contains 16 items, divided into four dimensions: withdrawal symptoms, social comfort, salience, and mood changes, which are widely used in mainland China (Xiong et al., 2012). Previous scholars did not clearly distinguish between smartphones and non-smartphones when studying mobile phone addiction. Kwon et al. went further in their research on phone addiction, explicitly targeting the smartphone theme and developing the Smartphone Addiction Scale (SAS) as well as the short version of the Smartphone Addiction Scale (SAS-SV) for adolescents. The SAS with 33 items contains six dimensions (daily-life disturbance, withdrawal, overuse, tolerance, positive anticipation, and cyberspace-oriented relationship). The Cronbach’s alpha of this scale is 0.967. The SAS-SV includes 10 items, and its internal consistency reliability is 0.91 (Kwon et al., 2013a,b). Su, a scholar from mainland China, considered that the definition of SAS scale for smartphones was not clear enough and the sample size was limited, therefore combining with the research on smartphone application (App) addiction to compile the Smartphone Addiction Scale for College Students (SAS-C), which has an alpha coefficient of 0.86 and contains 22 items and six dimensions (withdrawal behavior, salience behavior, adverse effects, social comfort, use of App, and renewal of App; Su et al., 2014).

Other researchers believe that PMPU is a non-addictive behavior. Toda et al. assuming that PMPU is not an addictive behavior but a dependent behavior. They regarded PMPU as the excessive use of mobile phones in the aspects of time and morality (Toda et al., 2004). Considering that excessive use of mobile phones would come with risks to people’s health, they began to pay attention to PMPU (Toda et al., 2004). In 2004, they developed the world’s first PMPU questionnaire with female college students as the research object, namely the cellular phone dependence questionnaire (CPDQ). The scale had an alpha coefficient of 0.86 and contained 20 items and six dimensions (Investment in mobile IP connection services, overuse of call services, anxiety about not being able to use mobile phones, lack of publicity in mobile phone use, emphasis on mobile phones, and requirements for others to hold mobile phones; Toda et al., 2004). Some scholars also believe that PMPU is a non-addictive behavior. For example, Lopez-Fernandez et al. indicated that other types of addictive behaviors should not be confused with smartphone addiction (Lopez-Fernandez et al., 2017). Although some phone users consider themselves addicted to their phones, the essence of their addiction is not the phone itself (Balakrishnan and Griffiths, 2019), but the user’s engagement through the phone, the drive to engage in the activity, and the satisfaction of participating in the activity (Jeong et al., 2016). Therefore, the problem is not phone addiction, but the problematic use of the phone, a maladaptive use of the smartphone that damages the user’s functionality (Hao et al., 2022). A large number of researchers have discussed the accuracy of the term PMPU from different perspectives, but no consistent conclusion has been reached so far.

Although the traditional definition of phone addiction has similarities to PMPU, the current severity of PMPU is much lower than that of addiction, so using the term “phone addiction” is inappropriate (Panova and Carbonell, 2018). In addition, the DSM-5 and ICD-11 did not formally define PMPU (American Psychiatric Association, 2013; World Health Organization, 2018). Hence the researchers chose to use the term “phone addiction” with caution (Roig-Vila et al., 2020; Hao et al., 2021). The subjects in this study are regular students, so it is appropriate to use the term PMPU. Previous studies have found that the detection rate of PMPU among students is relatively high. For example, the detection rate of PMPU among Spanish 12–18 year-old students is about 14.8% (De-Sola Gutiérrez et al., 2016), and the detection rate of PMPU among college students in mainland China is 13.8% (Zhan et al., 2020). Other studies have found that the level of PMPU would be higher year by year (Jun, 2019). What’s more, PMPU can lead to some adverse effects on students, such as learning distraction, anxiety sleep disorders, and even suicidal ideation and non-suicidal self-injury, etc. (Li et al., 2019; Arrivillaga et al., 2020; Yang et al., 2020; Jin et al., 2021; Troll et al., 2021). Therefore, how to restrain and reduce students’ PMPU has become a hot issue for scholars.

Researchers have discussed the formation mechanism of PMPU from neurophysiological, sociocultural, and individual psychological perspectives (Jamaluddin et al., 2020; Li et al., 2021; Hao et al., 2022; Liu Q. et al., 2022). In addition, some scholars have conducted related research from individual psychology, such as exploring the relationship between Academic Burnout (AB) and PMPU. AB, also known as academic boredom, learning burnout and student burnout (Olwage and Mostert, 2014; Feng et al., 2019; Shen et al., 2021). “AB” means the negative attitude and behavior of feeling bored with learning due to pressure or lack of interest in learning (Mao et al., 2015), mainly appeared as emotional exhaustion, cynicism and academic inefficiency (Kendall and Castro-Alves, 2018; Aguayo et al., 2019; Hao et al., 2022). At first, scholars did not pay more attention to AB but studied job burnout and gradually expanded to the field of learning. In 1981, Maslach and Jackson developed the first burnout inventory, the Maslach Burnout Inventory (MBI; Maslach and Jackson, 1981), containing three dimensions (emotional exhaustion, personal accomplishment, and depersonalization), to study burnout in human services professionals. Schaufeli et al. expanded the field of MBI’s research subjects, defined burnout more general, and, based on MBI, developed the Maslach Burnout Inventory-General Survey (MBI-GS; Schaufeli et al., 1996), which includes three dimensions: exhaustion, cynicism, and professional efficacy. With the research studied further, some scholars have proposed that burnout also appears among students, and students’ academic tasks can be understood as work tasks in the academic environment (Schaufeli et al., 2002; Shin et al., 2011). AB is the feeling of students exhausted from learning, with some features (cynical, detached, and incompetent) in the study. To study the burnout degree of students, based on MBI-GS Schaufeli et al. compiled the Maslach Burnout Inventory-Student Survey (MBI-SS), which contains three dimensions: exhaustion, efficacy, and cynicism (Schaufeli et al., 2002).

MBI has also been widely used in mainland China. For example, Lian et al. developed the Learning Burnout Scale (LBS) suitable for Chinese college students regarding MBI, which includes three dimensions, dejection, improper behavior and reduced personal accomplishment (Lian et al., 2005). The LBS has been widely used in mainland China. Wu et al. drew on Maslach’s Three-factor Theory to compile the Student Burnout Inventory, which is suitable for Chinese junior high school students. This scale is divided into three dimensions: exhaustion, cynicism, and reduced efficacy (Wu et al., 2007). Several years later, based on their research, primary and high school students were included in the discussion scope and Wu et al. compiled the Adolescent Student Burnout Inventory (ASBI) which is suitable for elementary to high school students. It contains three dimensions: exhaustion, reduced efficacy, and learning cynicism (Wu et al., 2010).

Scholars have conducted many studies on the relationship between AB and PMPU. Among them, some scholars believe that AB can positively predict PMPU, and there is a significant positive correlation between AB and PMPU (Shen et al., 2021; Hao et al., 2022). If students are in the state of AB for a long time, they will have mental health problems such as anxiety and depression (Cheng et al., 2020; Fiorilli et al., 2020), decrease of sleep quality (Yan et al., 2018), poor academic performance (Yune et al., 2018). Individuals with psychological symptoms, sleep disorders and academic difficulties are more likely to present PMPU (Hawi and Samaha, 2016; Chen et al., 2019; Hao et al., 2021; Wang et al., 2021b). This point supports the compensatory Internet use theory, which states that in negative life situations, individuals create incentives to use the Internet to alleviate negative emotions, compensating for problems in real life (Kardefelt-Winther, 2014). Due to the continuous development of mobile phone functions, mobile phones have become common tools for teenagers to surf the Internet (Liu et al., 2020). Students with AB tend to overuse mobile phones to get rid of emotional difficulties (Walburg et al., 2016; Hao et al., 2021). Besides, social bonding theory believes that if an individual realizes that the greater the expectations of the environment (such as school, family and society), the more important the environmental order is to him, and the stronger the connection between the individual and the environment, so the less problematic behavior occurred (Hirschi, 1969). However, if students have AB, their school engagement will be reduced, and their behavior tends to deviate from school norms and expectations, thus leading to problematic behaviors, such as PMPU (Zhu et al., 2015; Chen Y. et al., 2021). Not only can AB predict PMPU, but also PMPU can predict AB. Supposing that students have PMPU, their self-esteem, psychological capital and family intimacy will be reduced, and alexithymia, family conflict and life pressure etc. will be increased (Kim and Lee, 2012a; Mei et al., 2018; You et al., 2019; Chen H. et al., 2022; Lai et al., 2022). Students with higher psychological symptoms, interpersonal barriers and life pressure are more likely to produce AB (Kim and Lee, 2012b; Lin and Huang, 2014; Romano et al., 2019; Luo et al., 2020; Wang et al., 2021a). According to the job demands-resources model, low job resources and high job demands will lead to job burnout (Schaufeli and Bakker, 2004). Students’ excessive use of mobile phones will reduce their learning engagement, thus reducing work resources, leading to job burnout (Schaufeli et al., 2002). Therefore, students with PMPU will produce AB.

However, some scholars have inconsistent conclusions that AB does not predict PMPU, and the relationship between them is insignificant. First of all, it may be related to the happy experience that mobile phones can bring to users. No matter what kind of service experienced by mobile phones, the experiential activities will bring users more happiness and more durable (Dunn et al., 2011). The higher the happiness, the lower the AB (Lee and Yang, 2016). In the meanwhile, some learning apps also refer to this when developing, making learning apps more interesting. Learning apps will reflect the characteristics of games, which relieve the boredom during students’ learning process, then enhance students’ learning motivation (Deng and Shao, 2012). However, the students with higher learning motivation have lower AB (Yu X. et al., 2022). Secondly, it may be related to the fact that mobile phone use can improve students’ attention and memory. Compared with teenagers who do not use mobile phones, those who use mobile phones show higher levels of attention, processing speed of attention process, and short term memory (Lee et al., 2001, 2003; Smythe and Costall, 2003). While, individuals with higher levels of attention and memory are more likely to have higher academic performance (Perez-Lloret et al., 2013; Lee et al., 2014). And the higher the students’ academic performance, the lower their AB (Usán Supervía and Salavera Bordás, 2020). Besides, maybe it is related to the function of mobile media. Research shows that mobile phones have many media functions, such as search engines, learning websites, web browsing, applications, audio, video, and e-reading (Shao and Wang, 2020). They make it easier for students to obtain learning resources, playing a significant positive impact on students’ learning outcomes (Shao and Wang, 2020). So the students with better learning effects may have a lower level of AB (He, 2011).

In addition, the inconsistent results of previous studies may be due to the moderating variables affecting the relationship between AB and PMPU, such as measurement instrument, region, age, gender, and publication year. Therefore, we hypothesized that the moderating variables that have an impact on the relationship between AB and PMPU are (a) measurement instrument, (b) region, (c) age, (d) gender, and (e) publication year.

Measurement instruments may affect the reliability of meta-analysis findings. When analyzing the relationship between AB and PMPU, it can be found that the tools used by researchers are different. The following tools are mainly used to measure PMPU: MPATS designed by Xiong et al. (2012), SAS-C prepared by Su et al. (2014), MPAI compiled by Leung (2008), SAS designed by Kwon et al. (2013b) and other self-made PSU questionnaires, such as MPD-M (Wang, 2011). The following instruments are used primarily to measure AB: LBS compiled by Lian et al. (2005), ASBI prepared by Wu et al. (2010), and other self-made AB questionnaires, such as LBS-M (Hu and Dai, 2007). Different measurement tools have different theoretical basis, dimension construction, and the number of questions, which affect the relationship between AB and PMPU to a certain extent. Therefore, this study intends to analyze the moderating effect of measurement tools on AB and PMPU.

Regional differences may lead to significant differences in the relationship between AB and PMPU. The Chinese mainland can be divided into three major regions: east, center, and west (Lei and Cui, 2016). Previous studies have shown that there is a low correlation between AB and PMPU among students in central China (Nie, 2014), and a moderate positive correlation in both eastern and western regions (Cheng, 2021; Deng, 2021). While other researchers found that AB was moderately positively correlated with PMPU among students in eastern, central, and western China (He et al., 2022; Li C. et al., 2022). Therefore, this study hypothesized that the relationship between AB and PMPU is obviously different between different regions.

Differences in age may be related to the differences in the relationship between AB and PMPU. Some studies have found that there is a low positive correlation between AB and PMPU in senior high school students (Nie, 2014), and a moderate positive correlation in junior high school students and college students (Lu, 2017; Cheng, 2021). In addition, studies have shown that the AB of college, high school, and junior high school students are positively correlated with PMPU to a moderate degree (Wan, 2020; He et al., 2022). Therefore, this study will analyze whether there is a significant age difference between AB and PMPU.

The gender differences may cause distinct differences in the relationship between AB and PMPU. Previous studies have found that there is a low positive correlation between AB and PMPU in female students (Nie, 2014) and a moderate positive correlation in male students (Ge, 2013). What’s more, some researchers found that there was a moderate positive correlation between AB and PMPU in both male and female students (Zhang et al., 2019; Ma et al., 2020). Given this, the study will analyze whether there is a significant gender difference between AB and PMPU.

Finally, the publication year is a moderating variable that affects the relationship between AB and PMPU. Previous studies have shown that the correlation between AB and PMPU increases with the years (Zhang et al., 2019; Zhang F. et al., 2020; Ye, 2021). Other studies have shown that the correlation between AB and PMPU decreases over growing years (Wang, 2020; Liu et al., 2021; He et al., 2022). Therefore, this study will explore the differences in AB and PMPU among students in different years.

Thus, the relationship between AB and PMPU is still unclear. Maybe previous studies have used small samples, which caused the deviation. Therefore, this study adopted the meta-analysis method to explore the relationship between AB and PMPU by taking Chinese mainland adolescents as samples. Two research questions guided this study:

1. What is the size and direction of the relationship between AB and PMPU?
2. Will the study characteristics (measurement tools, region, age, gender, publication year) affect the relationship between AB and PMPU?

## Materials and methods

This meta-analysis would be carried out following the Preferred Reporting Items for Systematic reviews and Meta-Analyses (PRISMA) statement (Moher et al., 2009, 2015).

In order to increase transparencies and prevent unintended duplication of effort, the protocol of this meta-analysis was preregistered at the International Prospective Register for Systematic Reviews (PROSPERO) (CRD:42022347277).

### Literature search

**Figure 1:**
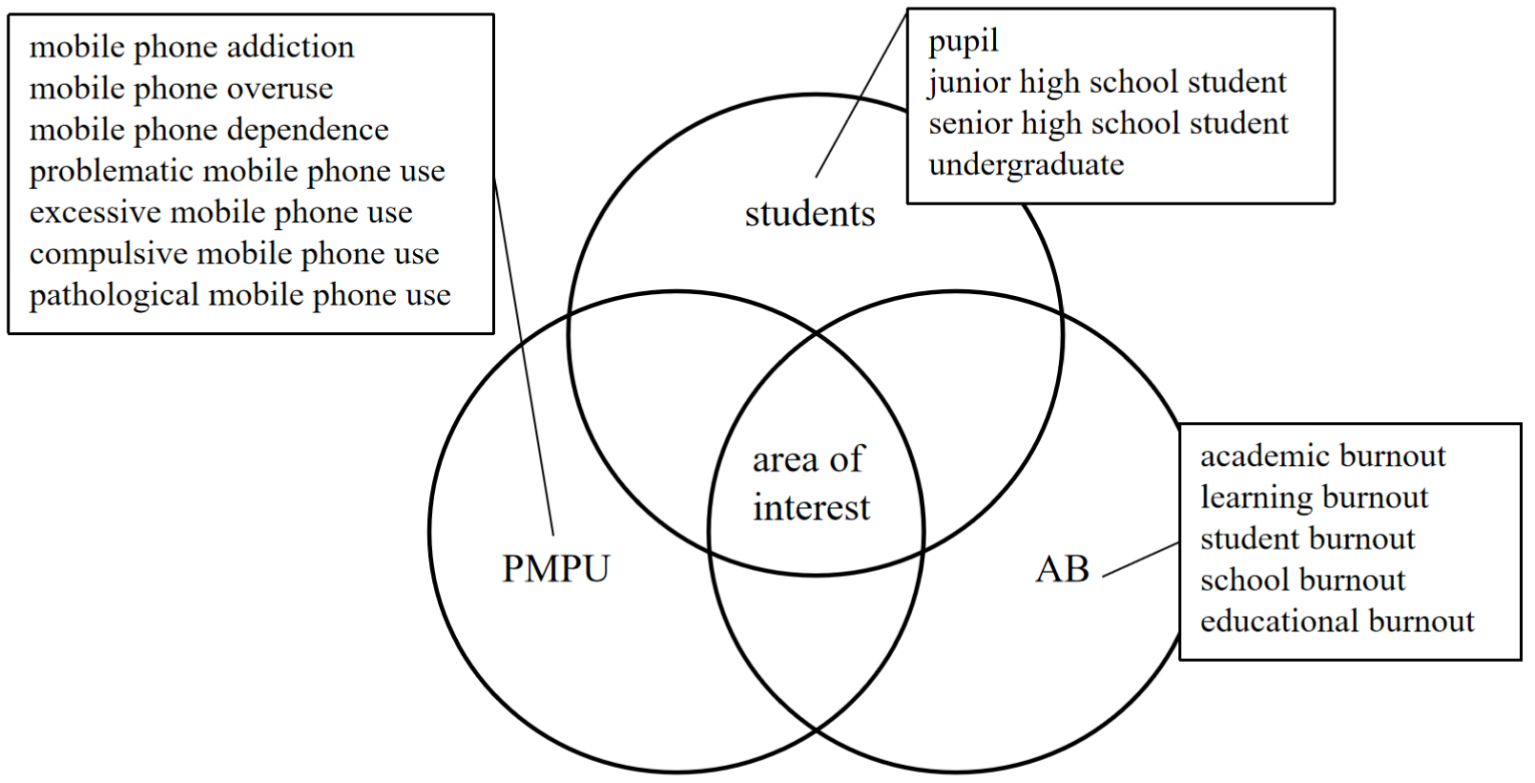
Diagram of search term clusters.

We used databases to search the related studies on AB and PMPU in Mainland China from January 2012 to November 2022, including CNKI, Wanfang Data, Chongqing VIP Information Co., Ltd. (VIP), Baidu scholar, ProQuest dissertations, SAGE Online Journals, Elsevier SDOL, Taylor & Francis, Springer, Web of Science, Google Scholar, EBSCO, PsycINFO, Medline, Scopus Database, PubMed Central, Embase, The Cochrane Library. In Figure 1 (Miller et al., 2018; Scott et al., 2018), the specific search terms are listed next to AB, PMPU and students. This paper studies the relationship between AB and PMPU of students.

**Figure 2:**
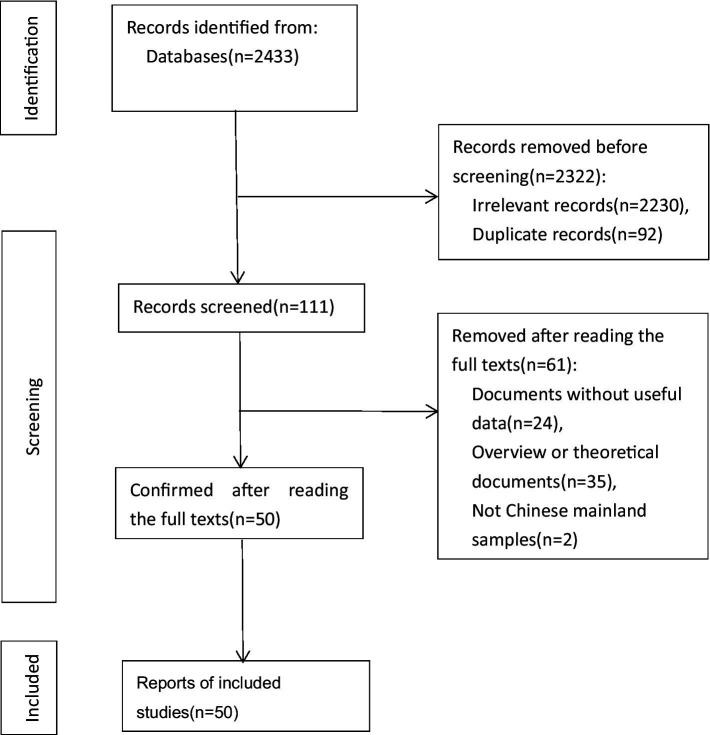
The PRISMA flow chart used to identify studies for detailed analysis of AB and PMPU.

The literature was screened according to the following criteria: (1) The AB scale and the PMPU scale were used simultaneously, and the Pearson product–moment correlation coefficient or the *t*-value and *f*-value that could be converted into r were reported; (2) The sample size is reported; (3) The subjects were students in mainland China, excluding other groups such as patients and criminals; (4) If data were repeatedly published, we only adopted professional academic journals. After excluding papers with no data, repeated publication, and no clear sample size, a total of 50 articles met the meta-analysis criteria. Figure 2 depicts the PRISMA flow chart of the systematic search.

### Coding variables

The collected articles were coded with features, including author’s name, publication year, regional distribution, literature type, age of subjects, sample size, the correlation coefficient between AB and PMPU, measurement tools of AB and PMPU, and the percentage of female students in the total population (see Table 1). Effect values were extracted according to the following criteria: (1) The correlation coefficient between AB and PMPU was included in the coding; (2) Independent samples were coded once; (3) When calculating the effect size of each category, each original datum appeared only once in each category to ensure the independence of the effect value calculation.

**Table 1:** Characteristics of the 50 studies included in the meta-analysis.

Name (year)	Journal	Region	Age	*N*	*r*	AB scale	PMPU scale	Female%
Bai et al. (2020)	General	Mixed	Younger	1794	0.168	Others	MPAI	49.00
Cao (2018)	General	Western	Undergraduate	193	0.348	LBS	SAS-C	54.92
Chen (2019)	Dissertation	Eastern	Younger	323	0.44	Others	SAS-C	33.13
Chen X. et al. (2021)	General	Eastern	Undergraduate	812	0.47	LBS	MPATS	65.52
Chen Y. et al. (2022)	General	Mixed	Undergraduate	1791	0.47	LBS	SAS-C	76.27
Cheng (2019)	Dissertation	Eastern	Undergraduate	673	0.51	LBS	MPAI	49.78
Cheng (2021)	General	Western	Undergraduate	885	0.402	LBS	MPAI	73.79
Cheng et al. (2018)	General	Eastern	Undergraduate	607	0.27	LBS	MPAI	60.96
Deng (2021)	General	Eastern	Younger	296	0.33	LBS	MPAI	34.46
Feng and Tao (2019)	General	Center	Undergraduate	704	0.466	LBS	SAS-C	54.26
Ge (2013)	General	Eastern	Younger	211	0.305	ASBI	MPATS	24.17
Gu et al. (2021)	General	Eastern	Undergraduate	389	0.481	LBS	MPATS	62.21
Hao et al. (2021)	General	Eastern	Undergraduate	748	0.348	LBS	SAS	76.20
Hao et al. (2022)	General	Eastern	Undergraduate	766	0.244	LBS	SAS	74.41
He et al. (2022)	General	Mixed	Younger	1,191	0.41	ASBI	MPAI	49.45
Hu (2022)	Dissertation	Mixed	Younger	576	0.47	Others	MPAI	51.56
Li et al. (2020)	General	Eastern	Undergraduate	825	0.215	LBS	MPAI	74.67
Li et al. (2021)	General	Eastern	Younger	2077	0.503	LBS	Others	86.52
Li B. et al. (2022)	General	Center	Younger	1,505	0.6	ASBI	MPAI	49.04
Li C. et al. (2022)	General	Mixed	Undergraduate	511	0.463	LBS	MPATS	77.50
Liang (2019)	Dissertation	Western	Younger	807	0.209	ASBI	MPATS	72.24
Liu Y. et al. (2019)	General	Center	Undergraduate	881	0.45	Others	MPATS	50.06
Liu et al. (2021)	General	Mixed	Undergraduate	323	0.471	ASBI	MPATS	78.02
Liu J. et al. (2022)	General	Western	Undergraduate	239	0.463	LBS	MPATS	63.60
Liu and Jin (2018)	General	Eastern	Undergraduate	397	0.52	LBS	MPAI	44.08
Lu (2017)	General	Eastern	Younger	1,010	0.363	ASBI	Others	50.00
Lu and Zhou (2019)	General	Eastern	Undergraduate	1,095	0.379	LBS	MPATS	95.07
Ma (2019)	Dissertation	Western	Younger	274	0.514	ASBI	SAS-C	60.95
Ma et al. (2020)	General	Eastern	Undergraduate	357	0.43	LBS	MPAI	90.20
Nie (2014)	Dissertation	Center	Younger	352	0.103	ASBI	MPAI	71.02
Nong (2022)	General	Western	Undergraduate	786	0.517	LBS	MPAI	62.34
Qin et al. (2020)	General	Western	Undergraduate	964	0.4	LBS	MPATS	55.60
Qu et al. (2017)	General	Eastern	Undergraduate	582	0.399	LBS	MPATS	77.32
Shen et al. (2021)	General	Center	Younger	631	0.46	Others	Others	46.75
Tian (2020)	General	Center	Undergraduate	300	0.4	LBS	MPATS	56.67
Wan (2020)	Dissertation	Mixed	Mixed	1,158	0.41	ASBI	MPAI	49.30
Wang (2020)	Dissertation	Mixed	Undergraduate	388	0.673	LBS	MPATS	55.67
Wang et al. (2022)	General	Western	Younger	2,260	0.39	Others	SAS	50.35
Wu et al. (2022)	General	Eastern	Undergraduate	883	0.474	LBS	MPATS	76.10
Ye (2021)	Dissertation	Center	Younger	312	0.548	ASBI	MPAI	50.64
Yu M. et al. (2022)	General	Eastern	Undergraduate	196	0.44	LBS	MPATS	85.20
Zhang et al. (2019)	General	Western	Undergraduate	239	0.348	LBS	MPAI	32.22
Zhang F. et al. (2020)	General	Eastern	Undergraduate	910	0.442	LBS	SAS-C	46.48
Zhang Y. et al. (2020)	General	Western	Undergraduate	635	0.338	LBS	SAS	61.42
Zhang et al. (2021a)	General	Western	Undergraduate	1,475	0.38	LBS	MPATS	67.73
Zhang et al. (2021b)	General	Western	Undergraduate	1,062	0.368	LBS	MPATS	60.26
Zhang and Miao (2021)	General	Eastern	Undergraduate	840	0.36	LBS	MPAI	56.79
Zhang and Shen (2015)	General	Eastern	Undergraduate	218	0.404	LBS	MPATS	46.33
Zhou (2021)	Dissertation	Eastern	Undergraduate	592	0.45	LBS	MPAI	78.04
Zhou et al. (2022)	General	Center	Undergraduate	1,445	0.431	LBS	SAS	50.00

### Effect size calculation

This study used the meta-analysis method of Person product difference correlation coefficient r to calculate the effect value (Borenstein et al., 2009). Fisher’s *Z* transformation was applied to *r*, and weights and 95% confidence intervals were calculated based on the sample size. Conversion formula: *Zr* = 0.5*ln[(1 + *r*)/(1−*r*)], *VZ* = 1/*n* − 3, *SEz* = sqrt(1/*n* − 3), where *Zr* represents the converted value of the corresponding *r*, *VZ* is the variance, and *SEz* is the standard error.

### Data processing and analysis

A homogeneity test is required to test whether each study result represents a sample estimate of the overall effect size. Firstly, the homogeneity test provides a basis for whether the outcome adopts a fixed effect model or a random effect model. If the test results showed that the effect values were homogeneous, the fixed effect model should be used. If the heterogeneity is considerable, the random effect model was selected. Secondly, the homogeneity test also provides the basis for analyzing the moderating effect, and the large heterogeneity indicates the existence of the moderating effect (Lipsey and Wilson, 2001).

## Results

### Effect size and the homogeneity test

Among the literature included in the meta-analysis of this study, 50 pieces of literature reflected the relationship between AB and PMPU, involving 38,488 subjects, with the sample size ranging from 193 to 2,260. Table 2 shows the homogeneity test of AB and PMPU in 50 independent samples, with *Q* statistic value of 591.365, *p* < 0.001, *I*^2^ = 91.714, indicating the heterogeneity of the included literature. This may be due to the use of different measurement tools, sources of subjects, and different sample sizes in the literature. That is, there may be a moderating effect. According to the method provided by Lipsey and Wilson, the included papers are highly heterogeneous and must be analyzed by random models (Lipsey and Wilson, 2001).

**Table 2:** Random model of the correlation between AB and PMPU.

*k*	*N*	Mean *r*	95% CI for *r*	Homogeneity test			Tau-squared			Test of null
				*Q* (*r*)	*p*	*I* ^2^	*Tau* ^2^	SE	Tau	*Z*-value
50	38,488	0.414	[0.384, 0.443]	591.365	0.000	91.714	0.015	0.004	0.121	24.272***

*p < 0.05, **p < 0.01, ***p < 0.001, the same as follows.

The stochastic model was used to analyze the correlation between AB and PMPU, and it showed that AB was significantly correlated with PMPU, with a correlation coefficient of 0.414, 95%CI [0.384, 0.443]. This relationship between AB and PMPU can be considered as a moderate correlation (Lipsey and Wilson, 2001). The Z-value of the relationship between AB and PMPU was 24.272, *p* < 0.001, indicating that the relationship between AB and PMPU was stable.

### Moderator analysis

As mentioned above, random effects models should also be used in moderating effects analysis. Meta-ANOVA analysis is suitable for analyzing the moderating effects of categorical variables, such as the type of measurement tool, subject group, regional differences, etc. In contrast, meta-regression analysis is suitable for analyzing the moderating effect of continuous variables, such as proportion of females and publication year.

#### Meta-ANOVA analysis

In order to further analyze the moderating effect of the relationship between AB and PMPU, Meta-ANOVA analysis was used to analyze the moderating effect of categorical variables (Table 3). In terms of region, the results of the homogeneity test (*Q* = 1.914, *df* = 3, *p* > 0.05) showed that region did not have a moderating effect on this correlation, and the relationship between AB and PMPU was not affected by region.

**Table 3:** Region and age and measures moderators of the association between AB and PMPU.

	*Q_between_*	*k*	*N*	Mean *r*	SE	95% CI for *r*		*Q_within_*
						LL	UL	
*Region*	1.914							
Eastern		22		0.402	0.004	0.363	0.440	161.643***
Center		8		0.443	0.014	0.353	0.524	117.658***
Western		12		0.390	0.004	0.346	0.433	64.951***
Mixed		8		0.449	0.019	0.346	0.541	198.392***
*Age*	0.469							
Undergraduate		34		0.421	0.003	0.393	0.449	225.522***
Younger		15		0.396	0.014	0.319	0.468	363.395***
Mixed		1		0.410	0.000	0.361	0.457	0.000
*Measures of AB*	0.241							
LBS		34		0.420	0.003	0.390	0.448	249.678***
ASBI		10		0.403	0.018	0.305	0.493	198.762***
Others		6		0.398	0.017	0.291	0.496	112.050***
*Measures of PMPU*	11.478*							
MPAI		18		0.400	0.012	0.330	0.466	370.697***
SAS-C		6		0.458	0.001	0.430	0.484	5.765
MPATS		18		0.427	0.005	0.384	0.469	125.346***
SAS		5		0.355	0.004	0.295	0.412	24.989***
Others		3		0.445	0.010	0.352	0.528	20.283***

In terms of age, the results of the homogeneity test (*Q* = 0.469, *df* = 2, *p* > 0.05) showed that age did not have a moderating effect on this correlation, and the relationship between AB and PMPU was not affected by age.

In terms of the measurement tool of AB, the results of the homogeneity test (*Q* = 0.241, *df* = 2, *p* > 0.05) showed that the measurement tool of AB did not have a moderating effect on this correlation, and the relationship between AB and PMPU was not affected by measurement tool of AB.

In terms of measurement tool of PMPU, the results of homogeneity test (*Q* = 11.478, *df* = 4, *p* < 0.05) showed that measurement tool of PMPU had a moderating effect on this correlation, and the correlation coefficients between AB and PMPU in MPAI, SAS-C, MPATS and SAS research were 0.400(95% CI = [0.330, 0.466]), 0.458(95% CI = [0.430, 0.484]), 0.427(95% CI = [0.384, 0.469]) and 0.355(95% CI = [0.295, 0.412]) respectively, which illustrates *r*_SAS_ < *r*_MPAI_ < *r*_MPATS_ < *r*_SAS-C_.

#### Meta-regression analysis

To examine whether continuous variables (gender and publication year) moderate the effect sizes between AB and PMPU, the *r* effect size was meta-regressed onto the percentage of female participants and publication year in each sample. In Table 4, meta-regression (Q_*Model*[1, *k* = 50]_ = 0.15, *p* > 0.05) demonstrated that the relation between AB and PMPU was not moderated by gender. Meta-regression (Q_*Model*[1, *k* = 50]_ = 6.87, *p* < 0.01) demonstrated that the relation between AB and PMPU was moderated by publication year. It means with the increase of publication year, the correlation coefficient between AB and PMPU also increases.

**Table 4:** Meta-regression analysis of gender and publication year.

Variables	Parameter	Estimate	SE	*Z*-value	95% CI for *b*	
					LL	UL
Female (%)	*β*_0_	−0.0469	0.1205	−0.39	−0.2830	0.1893
	*β*_1_	0.4687	0.0753	6.22	0.3211	0.6163
	*Q_Model_* (1, *k* = 50) = 0.15, *p* > 0.05					
Publication year	*β*_0_	0.0229	0.0087	2.62	0.0058	0.0400
	*β*_1_	−45.7433	17.6146	−2.60	−80.2672	−11.2193
	*Q_Model_* (1, *k* = 50) = 6.87, *p* < 0.01					

### Publication bias

**Figure 3:**
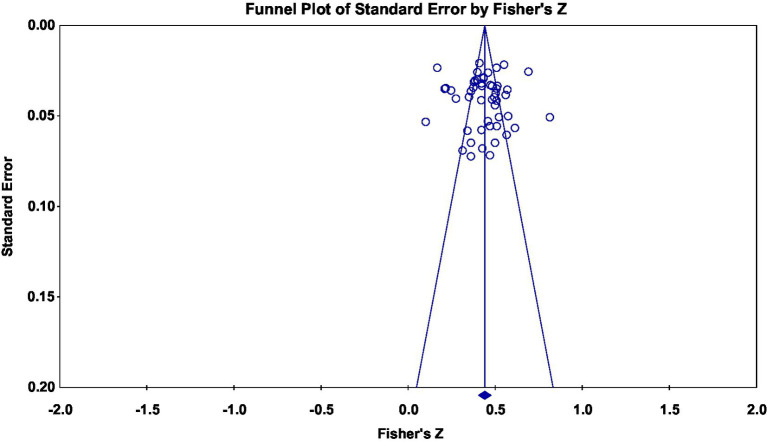
Funnel plot of effect sizes of the correlation between AB and PMPU.

To examine whether the results were biased due to effect sizes from various sources, a funnel plot was drawn, indicating that the 50 effect sizes were symmetrically distributed on both sides of the average effect size, and an Egger’s regression (Egger et al., 1997) revealed no significant bias (*t*_(48)_ = 0.214, *p* = 0.832 > 0.05). This result showed that in the overall correlation between AB and PMPU was stable in this study (Figure 3).

## Discussion

The results of the meta-analysis showed that there was a moderate positive association between AB and PMPU. The higher the AB, the higher the risk of PMPU, which is consistent with previous research (Qu et al., 2017; Shen et al., 2021). It supports the compensatory Internet use theory. According to this theory, individuals in negative life situations tend to use the Internet to compensate for their negative emotions (Kardefelt-Winther, 2014). Kardefelt-Winther, considering problematic Internet use from the perspective of compensation rather than forcing, thought that the problematic use of the Internet is a response based on positive or negative motivation, in which the individual’s response to negative life situations is the reason for compensatory Internet use, and the response can compensate for negative emotions through using the Internet to generate positive feelings (Kardefelt-Winther, 2014). Suppose students in the learning situation have negative emotions such as burnout. In that case, their learning desire will be reduced, resulting in a decline in academic performance, so they tend to use mobile phones excessively to alleviate the burnout, resulting in PMPU (Fiorilli et al., 2017; Wei et al., 2021). In addition, students who overuse mobile phones develop AB further (Zhen et al., 2020). It supports the job demands-resources model, which thinks that any type of working condition is composed of job demands and job resources, and the increase of job demands and the decrease of job resources will lead to job burnout (Schaufeli and Bakker, 2004). Job demands refers to the physical or mental effort made by individuals to work; job resources include the physical, psychological, social and organizational aspects that support and help people in their work (Schaufeli and Bakker, 2004). For students, their academic tasks actually is working tasks in an academic environment (Schaufeli et al., 2002). If students have PMPU, they will occupy a large amount of physical and psychological resources (such as personal energy, time and cognition) in using mobile phone, and less disposable resources engage in learning, resulting in low work resources, thus producing AB (Samaha and Hawi, 2016; Paloș et al., 2019). On the other hand, if students spend a lot of time on mobile phones, the less time they spent on study, they will not be able to keep up with the progress of study and academic performance will be decreased, gradually losing interest in study, eventually result in the emergence of AB (Qu et al., 2017). In addition, previous studies have proved that PMPU can have negative effects on health (anxiety, sleep disorders, and even suicidal ideation; Arrivillaga et al., 2020; Yang et al., 2020; Jin et al., 2021), which will consume students’ energy according to the severity, leading to low concentration in learning, then producing AB.

The positive relationship between AB and PMPU is moderated by the PMPU measures used. Specifically, SAS-C and MPATS had the higher impact on the relationship between AB and PMPU, MPAI and SAS lower. There are two possible reasons for this. Firstly, it may be related to cultural adaptation. For example, MPATS and SAS-C were developed by Chinese mainland scholars for Chinese mainland students, while MPAI was developed by scholars for students in Hong Kong, China, SAS was developed by scholars for students in South Korea. Therefore, MPATS and SAS-C can more accurately measure the PMPU of Chinese mainland students. Secondly, it may be related to whether the questionnaire is compiled for a specific learning stage. MPATS and SAS-C are developed for college students, while MPAI and SAS do not target specific study periods. Therefore, MPATS and SAS-C are more accurate in measuring college students, adjusting the relationship between AB and PMPU.

So far, studies have found that publication year moderates the relationship between AB and PMPU. This means that the relationship between AB and PMPU increases with increasing years. There are four possible reasons. Firstly, the report of CNNIC from 2012 to the latest 2022 shows that the number of mobile Internet users has increased from 420 million to 1.047 billion (CNNIC, 2013, 2022). It means that with the year’s progress, mobile phones are becoming more and more popular. According to the Internet availability hypothesis, availability has an essential impact on addictive behaviors (Mann, 2005; Su et al., 2019), which increases the likelihood of PMPU, thereby moderating the relationship between AB and PMPU. Secondly, research shows that during the COVID-19 epidemic, the use of digital technologies by people is on the rise (Montag and Elhai, 2020). Also, online learning will aggravate students’ AB (Liu and Cao, 2022). These factors will adjust the relationship between AB and PMPU. Thirdly, compared with the previous use of mobile phones, the 5G network is faster and mobile phones have more powerful functions. Through mobile phones people can communicate, learn, entertain, shop and so on (CNNIC, 2022), which will cause individuals to spend more time using mobile phones, thus adjusting the relationship between AB and PMPU. Fourthly, many studies have shown that AB increases over time (Lee, 2010; Kim et al., 2015), so publication year affects the relationship between AB and PMPU.

## Implications

From the study on the relationship between AB and PMPU, it implied that targeted measures can be taken to reduce students’ PMPU in the future. On the one hand, PMPU can be reduced by alleviating students’ AB. According to ecological systems theory, students, schools and families can make efforts together to alleviate students’ AB (Bronfenbrenner, 1992). First of all, from the perspective of students, it can improve students’ self-efficacy, self-concept, self-esteem and resilience to rebuild positive self-image, enhance participation and adaptability to have a higher level mental health (Mikaeeli et al., 2013; Luo et al., 2016; Safarzaie et al., 2017; Oyoo et al., 2018). And then, AB could be alleviated. Secondly, teachers can activate the intrinsic motivation resources of students by meeting their independent needs, so as to motivate and attract students (Reeve, 2006). Specifically, teachers can coordinate teaching activities according to students’ preferences, interests, enjoyment and choices, so as to alleviate students’ AB (Shih, 2015). Third, schools can provide professionals to reduce students’ AB, such as school health nurses, school doctors, school counselors, school psychologists, etc., to provide more professional help for students with learning difficulties (Kim et al., 2018). Finally, parents need to create a good family environment for their children. Students living in a warm, emotional environment can gain more support and understanding, resulting in a more positive attitude toward learning and less AB (Luo et al., 2020). Through the joint efforts of students, teachers, schools and parents, students’ AB can be alleviated and PMPU can be reduced.

On the other hand, positive coping strategies are needed to adopt for reducing students’ PMPU. The study found that students with psychological symptoms such as anxiety, depression and stress were more likely to get PMPU, while students with mild psychological symptoms were less likely to get PMPU (Augner and Hacker, 2012; Zou et al., 2019; Kong et al., 2021). Therefore, schools can regularly screen students with mental problems and provide professional mental health services for them (Liu R. D. et al., 2019; Ohrt et al., 2020). In addition, family members should also take measures to reduce the influence of mobile phones and other media on children. For example, parents monitor the time their children spend on their mobile phones, and parents communicate with their children about Internet contents to reduce the influence of media on their children. When these measures were associated with good parenting, the effect of PMPU prevention was more significant (Hefner et al., 2019). At the same time, communication between families and schools needs to be strengthened to educate students about the potential dangers of excessive cell phone use and guide them to set goals to control the frequency and duration of their phone use (Chun, 2018). These strategies may help students to use their phones in a healthy way.

## Limitations and future studies

This study applied Egger’s publication bias test to the meta-analysis results. It was revealed that the included studies had no obvious publication bias and that the meta-analysis results were stable. This indicates that, compared with the results of studies based on a single sample group, our results were more reliable, representative, and authentic. Future studies should explore the following aspects. Firstly, our data are based on self-reporting of the samples, suggesting that future researchers introduce other assessment methods to determine the relationship between AB and PMPU. Secondly, future research samples can be selected from a wider range of people, to improve the universality of research results. Thirdly, this meta-analysis only tested the moderating effects of the measurement instrument, region, age, gender and publication year. Future research can be analyzed from the perspective of other related moderator variables. Lastly, the researches included in this meta-analysis are mainly cross-sectional studies. Therefore, it is recommended to include longitudinal studies in future studies.

## Conclusion

We conducted a meta-analysis of data from 38,488 Chinese mainland students surveyed in 50 previous studies. The results showed a moderate positive correlation between AB and PMPU. That is, the higher the AB level is, the higher the PMPU level is. Conversely, the lower the AB level is, the lower the PMPU level is. The relationship is moderated by the measurement of PMPU and publication year. Specifically, firstly, the relationship between AB and PMPU is regulated by the measurement of PMPU. When the MPATS and the SAS-C were used as PMPU measurement tools, the correlation coefficients between AB and PMPU were higher, while when the MPAI and the SAS were used as PMPU measurement tools, the correlation coefficient between AB and PMPU were lower. Secondly, the relationship between AB and PMPU was moderated by publication year. When the publication year was used as a moderating variable, the relationship between AB and PMPU increased over the years.

## Data availability statement

The original contributions presented in the study are included in the article/supplementary materials, further inquiries can be directed to the corresponding author.

## Author contributions

SL designed and supervised the study, and did all statistical analyses. MX did the literature search and drafted the first version of the article. YZ and XW contributed to review and revision. All authors contributed to the article and approved the submitted version.

## Funding

This research was sponsored by the Project of Social Science Foundation of Xinjiang Uygur Autonomous Region (22CMZ018) and the Project of Center for Teacher Education Research in Xinjiang (ZK202232B) and the Project of Center for Higher Education Developmet Research in Xinjiang (ZK202283B) and the project of Doctoral Research Startup Fund at Xinjiang Normal University (XJNUBS201908).

## Conflict of interest

The authors declare that the research was conducted in the absence of any commercial or financial relationships that could be construed as a potential conflict of interest.

## Publisher’s note

All claims expressed in this article are solely those of the authors and do not necessarily represent those of their affiliated organizations, or those of the publisher, the editors and the reviewers. Any product that may be evaluated in this article, or claim that may be made by its manufacturer, is not guaranteed or endorsed by the publisher.
